# Hepatic Histotripsy: Jet Ventilation Decreases the Effect of Respiratory Motion in A Porcine Liver Model

**DOI:** 10.1007/s00270-025-04060-4

**Published:** 2025-06-04

**Authors:** J. Erik Winterholler, Meridith A. Kisting, Katrina L. Falk, Adrienne L. Kisting, Madeline S. Jentink, Jim K. White, Meghan G. Lubner, Paul F. Laeseke, Erica M. Knavel Koepsel, John F. Swietlik, J. Louis Hinshaw, Tatiana H. Ferreira, Lu Mao, Timothy McCormick, Min Cui, Fred T. Lee, Timothy J. Ziemlewicz

**Affiliations:** 1 Departments of Radiology, University of Wisconsin Hospitals and Clinics, 600 Highland Avenue, Madison, WI 53792 USA; 2 Biomedical Engineering, University of Wisconsin Hospitals and Clinics, 600 Highland Avenue, Madison, WI 53792 USA; 3 Urology, University of Wisconsin Hospitals and Clinics, 600 Highland Avenue, Madison, WI 53792 USA; 4 Biostatistics and Medical Informatics, University of Wisconsin Hospitals and Clinics, 600 Highland Avenue, Madison, WI 53792 USA; 5 Anesthesia University of Wisconsin Hospitals and Clinics, 600 Highland Avenue, Madison, WI 53792 USA; 6 Carle Illinois College of Medicine, 506 S Mathews Ave, Urbana, IL 61801 USA; 7 Department of Surgical Sciences, School of Veterinary Medicine, University of Wisconsin, 2015 Linden Drive, Madison, WI 53706 USA; 8 Department of Pathology, University Hospitals Cleveland Medical Center, 11100 Euclid Ave, Cleveland, OH 44106 USA

**Keywords:** Histotripsy, Ablation, Ultrasound, Anesthesia

## Abstract

**Purpose:**

To determine the efficacy of high-frequency jet ventilation (HFJV) to reduce the effects of respiratory motion on the resultant treatment zone during hepatic histotripsy in an in vivo porcine model.

**Materials and Methods:**

Non-tumor bearing swine (*n* = 6, 50.1 kg) underwent hepatic histotripsy with both HFJV and conventional ventilation (CV) in separate locations. Treatments were followed by contrast-enhanced magnetic resonance imaging (MRI) and necropsy. Treatment zone size (with emphasis on the cranial-caudal (CC) dimension), volume, and a survey for complications were conducted by MRI and at necropsy. Histopathology was performed to confirm complete cell kill in the treatment zone. Treatment precision was assessed by the width of the transition zone (TZ, zone of partial treatment) with a narrower TZ denoting increased precision.

**Results:**

Animals tolerated treatments with no adverse events. Treatment zones created during HFJV were less elongated than during CV (CC dimension during HFJV = 27.8 mm vs. CV = 32.3 mm, *p* = 0.04). Mean TZ width (treatment precision) was narrower in the HFJV group (HFJV = 4.1 mm, CV = 6.9 mm, *p* < 0.001), particularly at the cranial margin (HFJV = 4.0 mm, CV = 8.2, *p* = 0.018). Off-target treatments occurred in the chest wall (*n* = 2) and spleen (*n* = 1).

**Conclusion:**

High-frequency jet ventilation reduces the craniocaudal elongation of histotripsy treatment zones caused by respiratory motion and improves the precision of histotripsy compared to conventional ventilation in this animal model.

**Graphical Abstract:**

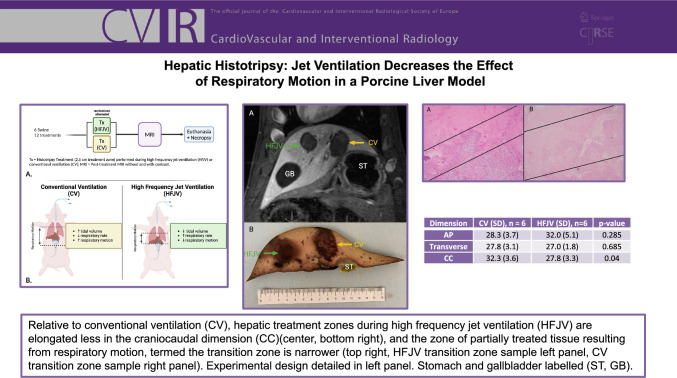

## Introduction

Histotripsy is a noninvasive, non-thermal, non-ionizing treatment modality recently approved for the destruction of liver tumors [1–3]. Histotripsy is characterized by complete cellular destruction of targeted tissue, highly precise treatment zones, tissue selectivity, and potentially beneficial immune effects which are under active investigation [4, 5]. A limitation of histotripsy is that respiratory motion can cause elongation of the treatment zone, particularly in the craniocaudal (CC) dimension [6–8]. Motion can also disperse the treatment effect by decreasing the time that the focal point dwells at a particular location, potentially making the treatment less effective and precise. To date, several strategies have been explored for reducing the effects of respiratory motion for histotripsy and related therapies, including respiratory gating, partial motion compensation with external robotics, and decreasing tidal volume during mechanical ventilation [9]. Limitations of these techniques include increased procedure times, increased complexity, and the potential for negative physiologic consequences such as hypoxia and respiratory acidosis/alkalosis.

High-frequency jet ventilation (HFJV) is a form of mechanical ventilation which employs rapid application of low tidal volume inspiratory jets followed by passive exhalation [10]. Prior work has demonstrated that HFJV results in less respiratory motion of the upper abdominal organs than conventional mechanical ventilation [11]. HFJV has historically been used for barotrauma risk reduction in neonates, ventilation during rigid bronchoscopy, and for otolaryngologic surgery [12–14]. More recently, HFJV is increasingly utilized during thoracic and abdominal image-guided locoregional therapies as minimization of respiratory motion with HFJV improves the accuracy of needle placement during ablation and precision of external energy delivery during stereotactic body radiation therapy (SBRT) [15–17].

The purpose of this study is to determine if the use of jet ventilation during hepatic histotripsy results in treatment zones with less elongation in the CC dimension when compared to conventional ventilation (CV) in a live porcine model. A secondary goal is to determine whether HFJV can improve the precision of histotripsy by decreasing the zone of partially treated liver tissue surrounding the treatment zone.

## Materials and Methods

### Experimental Design (Fig. 1)

**Fig. 1:**
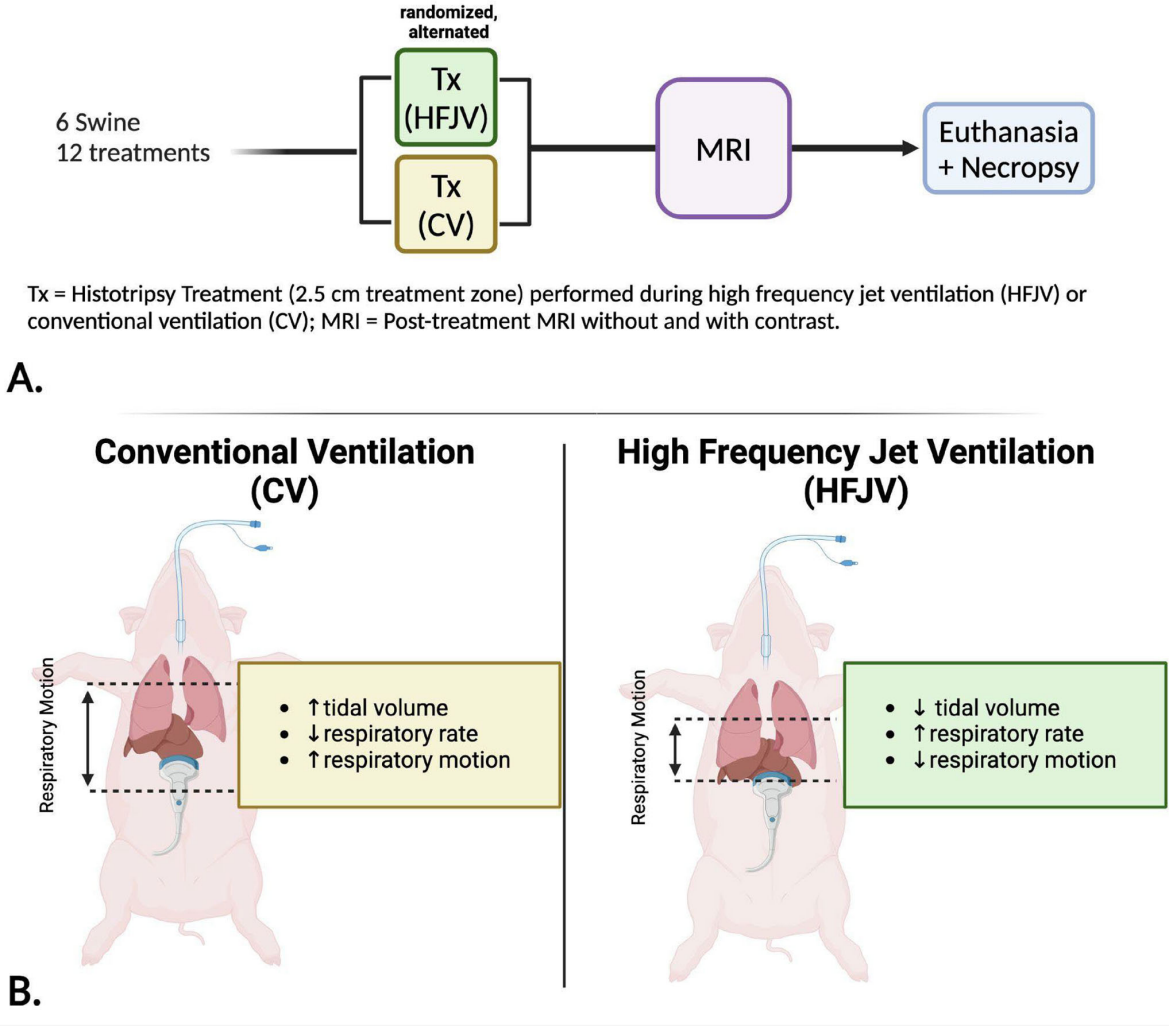
Experimental design comparing CV and HFJV for use during histotripsy

This study was approved by the Institutional Animal Care and Use Committee. Six healthy female swine (mean weight 50.1 kg, Arlington, Wisconsin, USA) were used for this study. Each animal underwent two identical histotripsy treatments in the liver (lobe randomized, alternated) with different ventilation strategies: 1) CV and 2) HFJV. Following completion of treatments, MRI was performed and animals euthanized using 4 mEq/kg supersaturated KCl (EMD Millipore Corp., Massachusetts USA) followed by necropsy. Livers were harvested and representative sections sent for histologic preparation with attention to the transition zone between normal and treated liver.

### Anesthesia Protocol

Pigs were premedicated with Telazol (Zoetis, Parsippany Troy Hills, New Jersey, USA) and Xylazine IM (Dechra Veterinary Products, Overland Park, Kansas, USA). Following intubation, isoflurane in 100% oxygen was used for anesthesia maintenance during CV. Pigs were mechanically ventilated with a tidal volume of 8–10 mL/kg and peak inspiratory pressure of 15–20 cm H2O for CV. The animals were maintained on CV throughout the study with the exception of the HFJV treatment arm.

High-frequency jet ventilation was performed as previously described [16, 18]. The following drugs were used to maintain anesthesia and paralysis: Ketamine (Dechra Veterinary Products, Overland Park, Kansas, USA), propofol (Pfizer Labs, New York City, New York, USA), dexmedetomidine (Dechra Veterinary Products, Overland Park, Kansas, USA), and vecuronium (AuroMedics Pharma, Windsor, New Jersey, USA). The jet ventilator (Monsoon III, Acutronic Medical Systems, Switzerland) was set at a driving pressure of 14 psi with a rate of 120 cpm and inspiratory time of 35%, delivered with 100% oxygen and climatization set at 4.

### Histotripsy Treatment

Two spherical treatment prescriptions with a diameter of 2.5 cm were created in each median lobe of the porcine liver: One with CV and one with jet ventilation. The laterality of treatment and whether conventional or jet ventilation treatment was performed first was prospectively randomized and then alternated. Treatments were performed as previously described [19]. In brief, a prototype histotripsy system (VortexRx, HistoSonics, Minnesota, USA), consisting of a dedicated 1-MHz therapeutic transducer and a coaxially aligned 3-MHz phased array diagnostic US probe (BK Medical, Massachusetts USA), was used to select and treat the planned area of liver. Due to thin hepatic lobes in swine, treatment zones overlapping into the chest or abdominal wall did occur to ensure two treatments could be performed in each animal. Additionally, thermal safeguards of the system were bypassed to ensure consistent treatment times for each zone. This bypass can lead to damage in the chest and abdominal wall, which is reported for completeness, though not germane to the study.

### Imaging

Post-histotripsy magnetic resonance imaging (MRI) was performed before and after administration of gadobenate dimeglumine at 0.1 mmol/kg (Bracco, Milan, Italy) within 2 h of the completion of the histotripsy treatments. Images were analyzed in consensus by two radiology residents (postgraduate years 2 and 3) and an abdominal imaging fellowship trained faculty radiologist with 15 years of experience. Treatment zones were measured in the craniocaudal (CC), transverse, and anteroposterior (AP) planes. Treatments extending across fissures were measure by addition of the treatment zone diameter perpendicular to the liver surface in each lobe. Treatment zone volume estimation was calculated using an ellipsoid formula. All images were assessed for complications and any signs of off-target damage.

### Pathology

Focused necropsy was performed after euthanasia. The peritoneal cavity was inspected for off-target treatments including the spleen, kidney, bowel and body/chest wall. Porcine livers were dissected from the porta hepatis, and the superior margin was marked with India ink. Where applicable, other intra-abdominal organs and portions of chest/body wall were also harvested. All collected organs were fixed in 10% buffered formalin.

Following fixation, the treatment zones were identified and samples of the transition zone (TZ), defined as the distance between completely treated and normal tissue, were collected from six locations for each treatment: cranial, caudal, anterior, posterior, left lateral, and right lateral [20]. These samples were placed in individual cassettes (Sakura Tissue-Tek VIP, California, USA), embedded (Leica EG 1160, Illinois, USA), and stained with hematoxylin and eosin. A pathologist with 5-year experience evaluated the samples and measured the partially treated TZ.

### Statistical Analysis

The sample size was based on results from previous animal studies of histotripsy, with the goal of minimizing the number of live animal subjects [6, 21]. A paired *t* test was used to compare the mean measurements of HFJV and CV diameters on post-procedural MRI, and TZ dimensions from the histopathologic analysis. A *p*-value of < 0.05 was considered statistically significant. All statistical analyses were performed using R (R-project.org) by a biostatistician.

## Results

### Histotripsy Procedure

The technical success rate of histotripsy treatment was 100% with all 12 liver treatments completed successfully, defined by visualization of a treatment zone in the liver on imaging and at pathology. All six animals tolerated the procedure without significant adverse events.

Treatment time was 13 min 23 s in all cases.

### Treatment Zone Appearance and Size

**Fig. 2:**
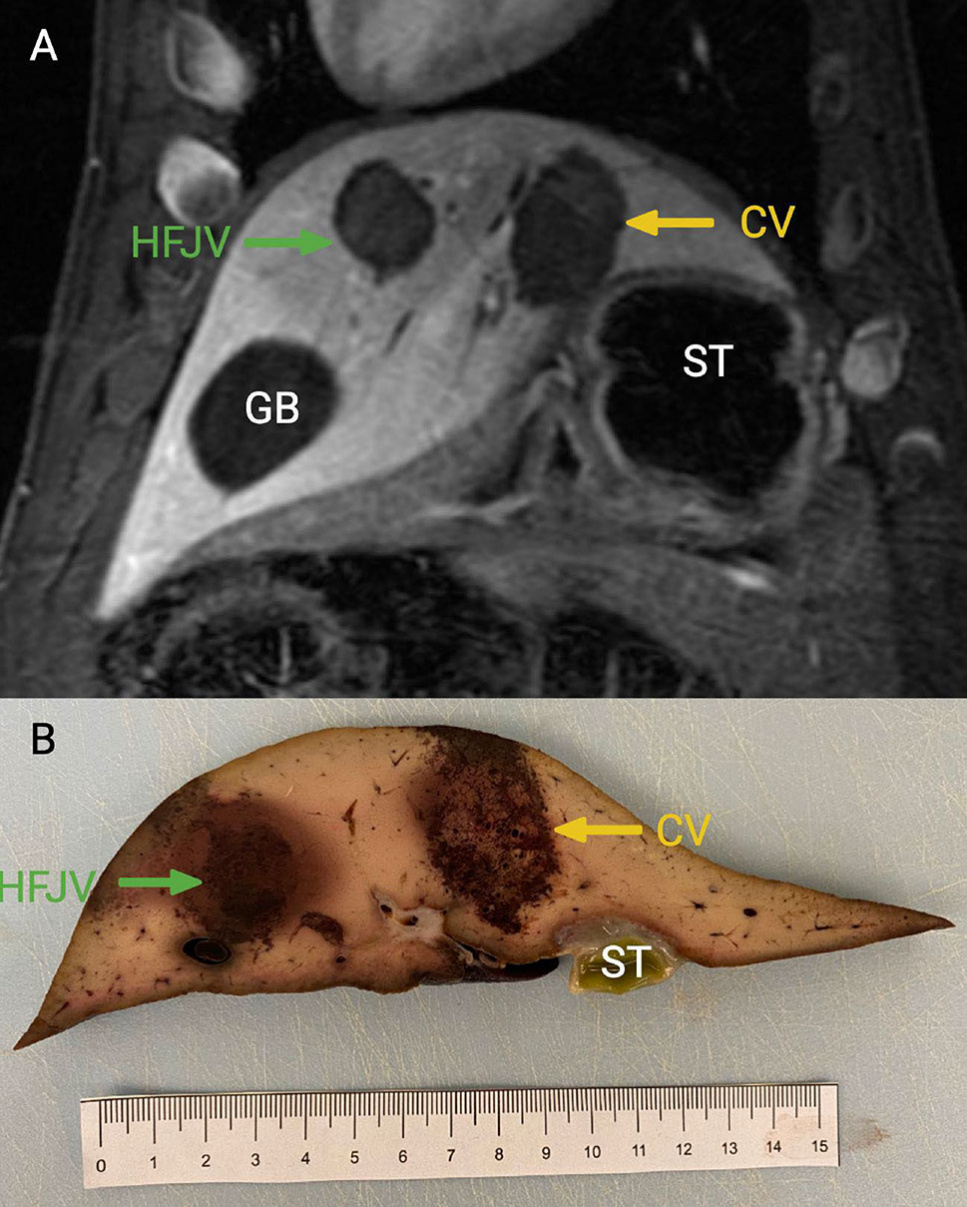
Treatment zone elongation in the craniocaudal dimension. Treatment zone performed during high-frequency jet ventilation (green arrow) and conventional ventilation (yellow arrow) on both contrast-enhanced MRI (panel A) and gross pathology (panel B). Stomach and gallbladder labeled (ST, GB). Craniocaudal dimensions for treatment zones pictured subject: HFJV = 32 mm, CV = 38 mm

The MRI appearance of hepatic histotripsy treatment zones was similar to previously described well-demarcated spherical-to-ovoid with high intrinsic signal intensity on T1-weighted images, minimally increased signal on T2-weighted images compared to background liver, and lack of enhancement after the administration of contrast [6, 7](Fig. 2).

The mean CC measurement of all treatment zones was 30.1 mm (*p* < 0.001 vs prescription). When subdivided by ventilation technique, the CC measurement was shorter and closer to prescribed for HFJV compared to the CV group (27.8 mm HFJV vs. 32.3 mm CV, *p* = 0.04, Table 1). The difference in the anteroposterior and transverse dimensions was not significantly different between HFJV and CV (Table 2).

**Table 1:** Treatment zone sizes vs prescribed

Dimension	Ventilation type	Prescribed size (mm)	Mean size (mm [SD])
AP	CV	25	28.3 (3.7)
	HFJV	25	32 (5.1)*
	ALL	25	30.2 (4.6)*
Transverse	CV	25	27.8 (3.1)
	HFJV	25	27 (1.8)*
	ALL	25	27.4 (2.5)*
CC	CV	25	32.3 (3.6)*
	HFJV	25	27.8 (3.3)
	ALL	25	30.1 (4)*
AP/Transverse/CC	CV	25	29.5 (3.9)*
	HFJV	25	28.9 (4.1)*
	ALL	25	29.2 (3.9)*

(*AP* anteroposterior, *CC* craniocaudal, *CV* conventional ventilation, *HFJV* high-frequency jet ventilation, * denotes statistical significance vs. prescribed)

**Table 2:** Comparison of treatment zone sizes (mm) by ventilation modality

Dimension	CV (SD), n = 6	HFJV (SD), n = 6	p-value
AP	28.3 (3.7)	32.0 (5.1)	0.285
Transverse	27.8 (3.1)	27.0 (1.8)	0.685
CC	32.3 (3.6)	27.8 (3.3)	0.04

(*AP* anteroposterior, *CC* craniocaudal, *CV* conventional ventilation, *HFJV* high-frequency jet ventilation) Note: Prescribed diameter = 25 mm in all dimensions

**Fig. 3:**
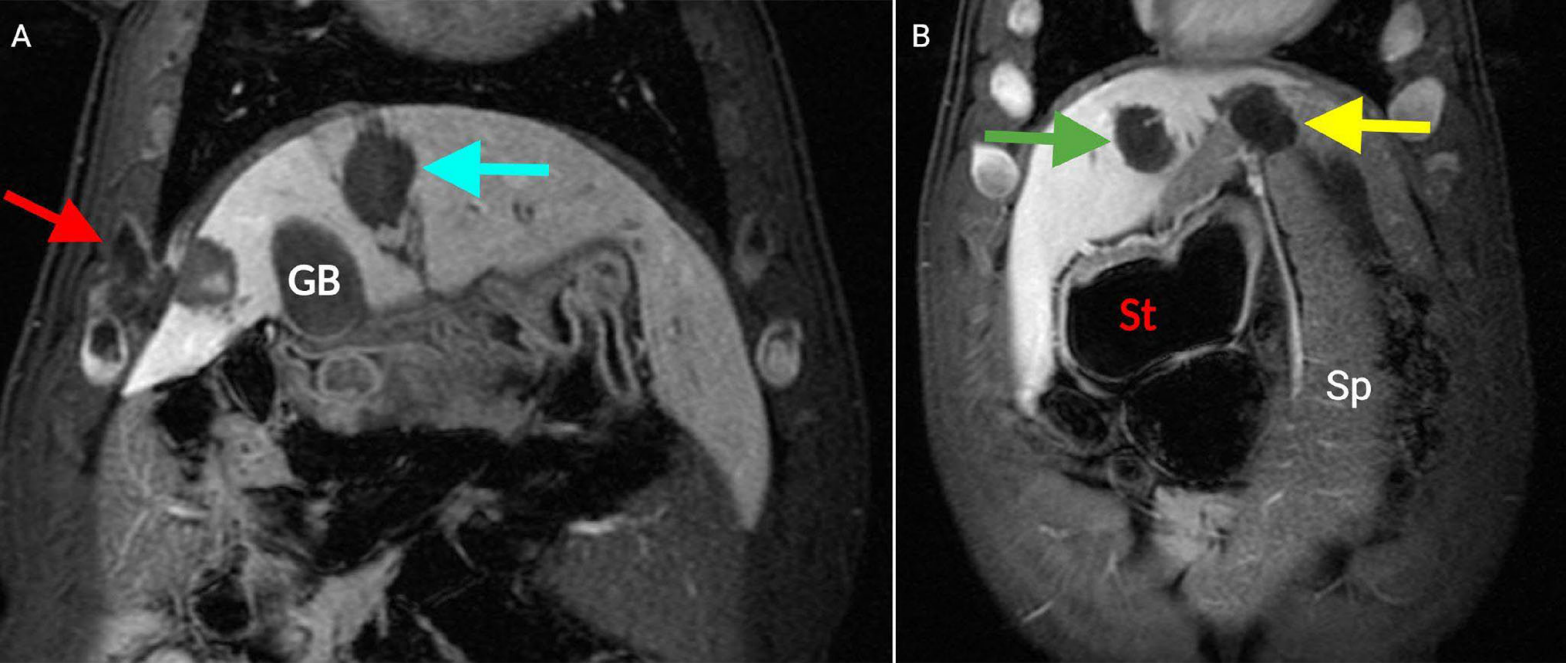
Contrast-enhanced T1-weighted MRI of off-target treatments in the coronal plane. **A**: Treatment extending into the chest wall (red arrow) vs. normal intrahepatic treatment (blue arrow). **B**: Splenic (yellow arrow) vs. hepatic treatments (green arrow). The spleen is immediately contiguous with the liver and difficult to distinguish by ultrasound in pigs. GB-gallbladder, St = stomach, Sp = spleen

**Fig. 4:**
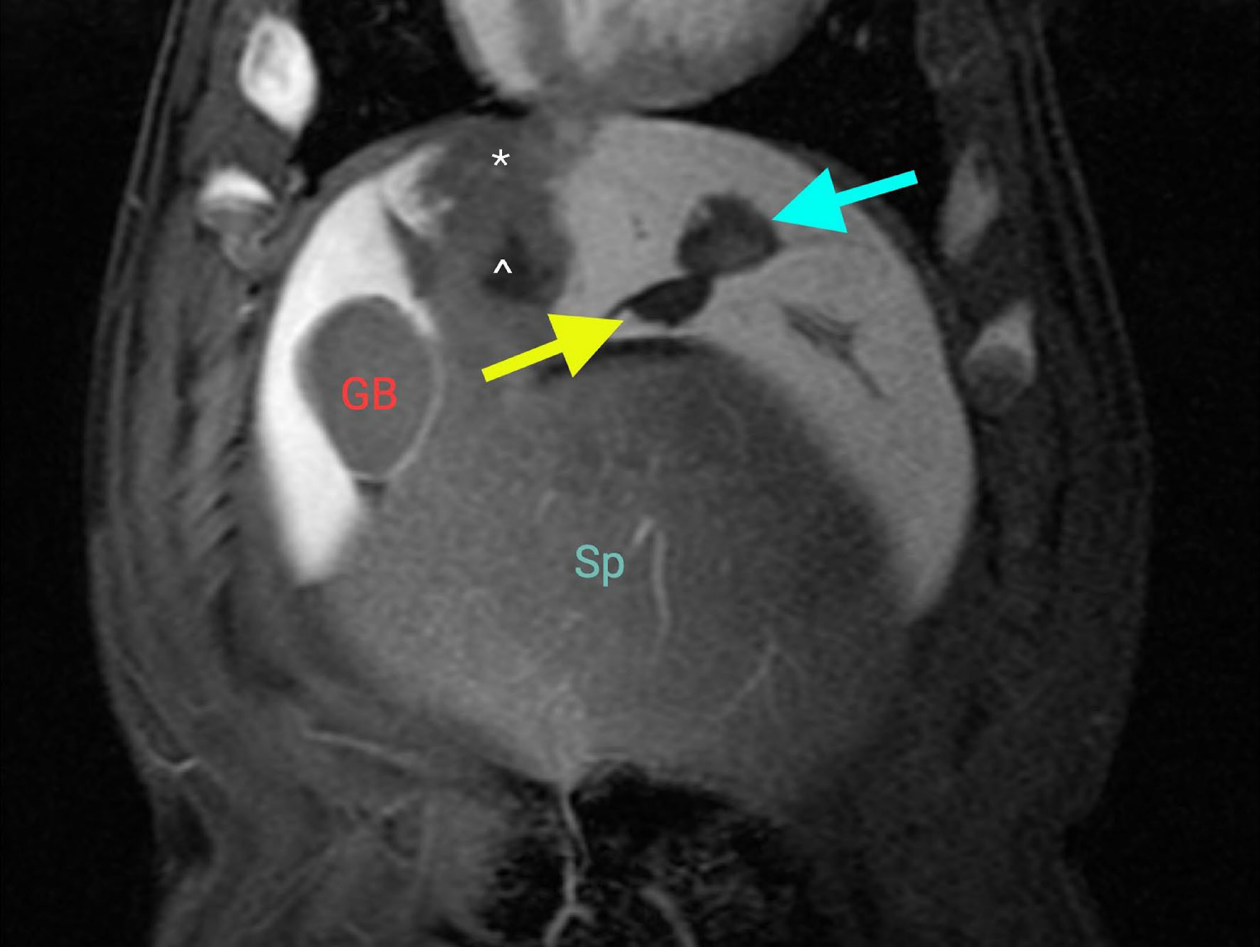
Transfissural treatment resulting in a separated treatment zone. Porcine liver lobes are divided by fissures not visible by ultrasound. This divided treatment zone across a fissure (upper treatment zone blue arrow, lower treatment zone yellow arrow) was caused by subject motion between the time of treatment and post-treatment MRI scan. Also noted on the scan is a perfusion defect (*) peripheral to the other treatment (^) in the animal, a reported finding on immediate post-procedure histotripsy imaging [2, 22]. Spleen (SP), gallbladder (GB)

The overall mean diameter of histotripsy treatment zones was 29.2 mm (*p* < 0.001 vs. prescribed (25 mm)). Divided by ventilation technique, the HFJV mean treatment zone diameter was 28.9 mm and the CV mean treatment zone diameter was 29.5 mm (both *p* < 0.001 vs prescription). Several areas of off-target damage were noted by MRI, including extension of the treatment zone into the chest wall (*n* = 2) and spleen (*n* = 1) (Fig. 3). Transfissural treatments were also noted in several subjects (Fig. 4).

### Gross and Microscopic Pathology, Including Transition Zone Measurements (Table 3)

**Table 3:** Mean transition zone measurement on histopathology at each treatment margin

Margin	CV mean in mm (SD), *n* = 6	HFJV mean in mm (SD), *n* = 6
Cranial	8.2 (2.6)	4 (1.5)*
Caudal	6.2 (4.1)	3.3 (0.8)
Anterior	8.5 (3.7)	3.8 (1.5)
Posterior	6.3 (2.7)	4 (1.3)
Left	5.8 (1.8)	4.2 (1.3)
Right	6.5 (3.4)	5.2 (1.3)
All	6.9 (3.1)	4.1 (1.3)*

(*CV* conventional ventilation, *HFJV* high-frequency jet ventilation). Asterisk (*) denotes statistical significance (*p* < 0.05)

Partial extension of treatment zones into the chest wall (*n* = 2) and spleen (*n* = 1) were confirmed at necropsy. The microscopic appearance of hepatic treatment zones was consistent with complete cellular destruction within the treatment zone, surrounded by a thin rim of partially treated tissue (transition zone). Both gross and microscopic descriptions were concordant with prior descriptions [19].

**Fig. 5:**
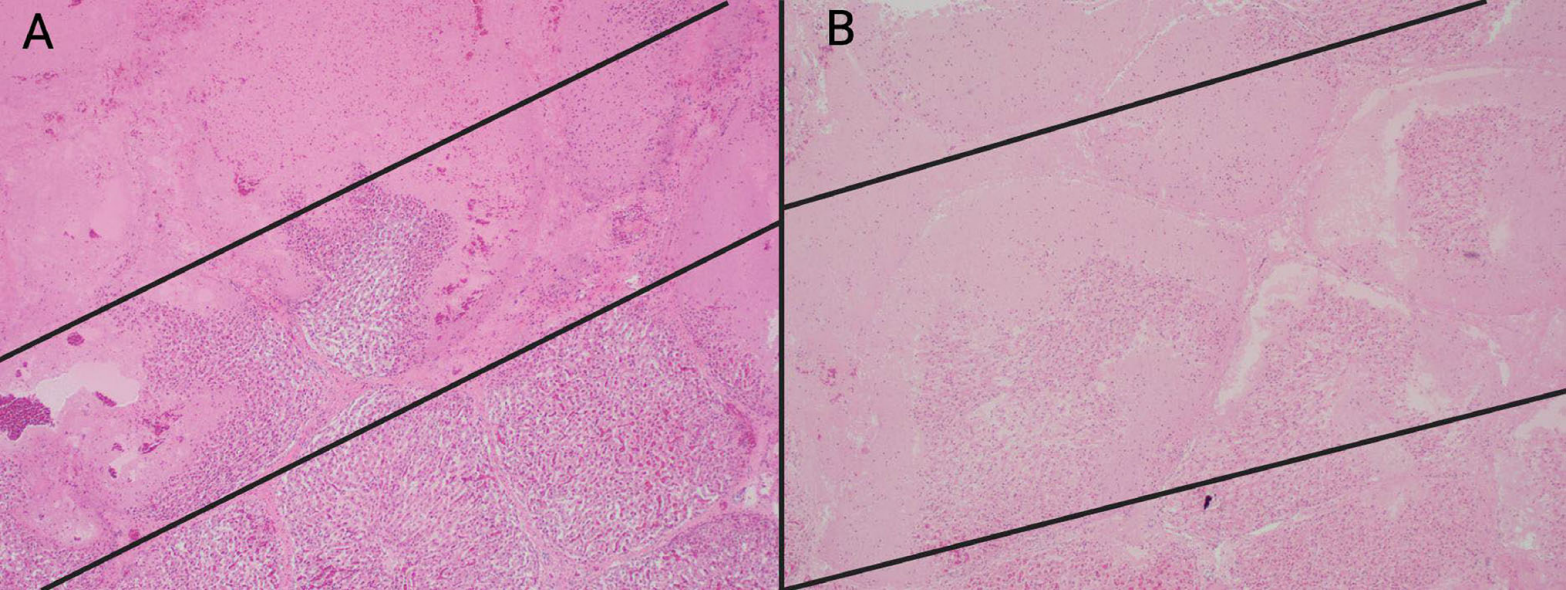
Transition zone between completely treated and normal liver. Hematoxylin and eosin slides of a representative transition zone margin for **A** a high-frequency jet ventilation zone and a **B** conventional ventilation treatment zone at 40 × total magnification. Note that at the same magnification, the high-frequency jet ventilation transition zone is narrower. The average transition zone was 4.1 mm in the high-frequency jet ventilation group and 6.9 in the conventional ventilation group

The mean transition zone measurement was 5.5 mm across all samples (Table 3). Overall, HFJV transition zones were narrower than CV (HFJV = 4.1 mm, CV = 6.9 mm, *p* < 0.001, Table 3, Fig. 5). When comparing individual sample locations, transition zones were significantly narrower at the cranial margin for HFJV when compared to CV (4.0 mm HFJV, 8.2 mm CV, *p* = 0.018). In the remaining locations, the transition zones were slightly more narrow for HFJV compared to CV, but this difference did not meet statistical significance.

## Discussion

The results of this study demonstrate that the use of jet ventilation during histotripsy results in a significant decrease in elongation of the treatment zone in the craniocaudal direction by ~ 5 mm. A secondary finding was that the transition zone between treated and untreated tissue was decreased with jet ventilation, resulting in more precise treatments.

The clinical significance of reducing distortion of a prescribed treatment zone is well recognized [23, 24]. By closely conforming to the pre-treatment prescription, collateral damage to structures above and below the intended treatment zone such as the diaphragm, lung, and hepatic hilar structures can be avoided. Interestingly, in this study, treatment dimensions in all planes were slightly larger than prescribed, even for HFJV subjects. This is an expected finding given that the use of jet ventilation is still associated with minimal but perceptible respiratory motion resulting from application of high-frequency low-volume air jets, but this motion is far less than with conventional ventilation [16].

A second key finding in this study is a decrease in the band of partial treatment (transition zone), particularly at the cranial margin of the treatment zone. Narrower transition zones are desirable due to a reduction in the width of partially treated tissue that may contain viable tumor cells [20]. With all heat-based ablation modalities, there is a thermal gradient within the treatment zone with the highest temperatures located adjacent to the probe and exponential cooling toward the periphery, resulting in a transition between non-viable and viable tissue. Histotripsy is different in that there is no thermal gradient, and in the absence of motion, all points within a prescribed treatment zone receive an equal number of ultrasound pulses (i.e., dose). However, respiratory motion can create an unequal distribution of histotripsy pulses throughout the treatment zone due to varying dwell times of the focal point. In particular, the cranial and caudal margins are vulnerable to undertreatment due to unevenness of the respiratory cycle with less time at the extremes of inspiration and expiration. HFJV appears to reduce this effect by reducing overall liver motion, resulting in narrower transition zones at the borders of the treatment prescription compared to conventional ventilation. Interestingly, there was no significant difference in transition zone at the caudal margin which most likely relates to the small sample size, though could also relate to the consistent location of the caudal treatment margin during expiration as compared with the more variable location of the cranial margin during inspiration.

All external beam therapies such as SBRT and thermal HIFU suffer from the effects of respiratory motion due to the lack of fixation of the energy source in relationship to the liver. While hepatic motion during respiration is complex and multidimensional, most of the motion is in the craniocaudal plane [6, 25]. To date, multiple strategies have been tested to minimize respiratory motion and its deleterious effects on external beam therapies. Respiratory gating was among the first methods to be trialed, though its use in histotripsy is limited due to an undesirable increase in the overall treatment time [26–29]. Respiratory gating also suffers from discrepancies between surrogate signals of respiration and the actual respiratory cycle, resulting in a decrease in precision [30]. Recently, external motion compensation with a robotic arm has been trialed with boiling histotripsy, though its application is complex due to the need for advanced robotics and highly accurate tracking of respiration during treatment [9]. Alteration of the treatment zone shape is an alternative passive method to account for CC motion by using a “flattened” prescription that when elongated by motion results in a more spherical shape [6]. However, changing the treatment zone shape in the CC dimension does not alter the transverse or AP dimensions, and does not account for a decrease in focal point dwell time at each treatment point. Thus, this method will likely be associated with a varying histotripsy dose throughout the treatment zone and potentially decreased precision (i.e., widening of the transition zone of incompletely treated tissue).

HFJV is a straight-forward approach to reduce respiratory motion that has been in use for more than 30 years [31]. There are now numerous broadly utilized clinical applications and data to support the safety and effectiveness of HFJV for existing and emerging therapies [12, 13]. HFJV has been shown to decrease liver motion and is well tolerated for percutaneous ablation procedures, even with prolonged anesthetic times [11, 16, 31]. As histotripsy becomes a more widespread clinical modality, HFJV is well suited for rapid integration due to existing regulatory approvals, readily available commercial devices, and growing experience with its use in the anesthesia community. This is also the first respiratory motion compensation technique explored with cavitation-cloud histotripsy and thus comparison with other methods cannot yet be made.

This study has several limitations. First, there are important anatomic differences between the porcine model and humans. For example, the human liver has relatively thick hepatic lobes with a broad plane of attachment, whereas the porcine liver has thin and independently mobile lobes separated by deep fissures. Thus, human and porcine livers will move and deform differently during ventilation making direct translation of treatment diameters and volumes problematic. However, the overall decrease in CC motion between species should still apply. The thin multilobar nature of the porcine liver also increases the chances of extending treatments into the body wall as well as treating across fissures which can separate the treatment zone at the time of necropsy, but this effect can be accounted for with simple measurement techniques. Another relevant anatomic difference between pigs and humans is the large porcine spleen which is often in contact with the underside of the liver. Because the ultrasound appearance of the spleen and liver is similar in pigs, treatments extending into the spleen were not surprising. Since both are cellular parenchymal organs that respond similarly to histotripsy, this limitation is not material to the results of this study [32]. In terms of other motion mitigation strategies, comparisons with techniques such as respiratory gating and selective intubation with bronchial blockade were not performed due to the lack of standardization of the technologies/techniques and costs/complexities in applying these techniques to an animal model. Lastly, the sample size was limited due to the desire to decrease the number of live animal subjects, and thus statistical significance was not met for some of the individual transition zone measurements. However, the sample size was sufficient to confirm the most important overall finding of narrower transition zones for HFJV vs. CV.

## Conclusion

High-frequency jet ventilation is increasingly used to minimize motion of abdominal organs during percutaneous and external beam therapies. The combination of HFJV and histotripsy appears to decrease cranial-caudal elongation and increase the precision of treatment zones in this large animal model.

## Data Availability

Figures were created with BioRender.com under license numbers LA27FDATCU, KX27FDAG0U, CV27FDANFA, PQ27FDAZQU, and ND27FDB411.

## References

[1] Wah TM, Pech M, Thormann M, Serres X, Littler P, Stenberg B, et al. A multi-centre, single arm, non-randomized, prospective european trial to evaluate the safety and efficacy of the histosonics system in the treatment of primary and metastatic liver cancers (#HOPE4LIVER). Cardiovasc Intervent Radiol. 2023;46:259–67. 36380155 10.1007/s00270-022-03309-6 PMC9892119

[2] Vidal-Jove J, Serres X, Vlaisavljevich E, Cannata J, Duryea A, Miller R, et al. First-in-man histotripsy of hepatic tumors: the THERESA trial, a feasibility study. Int J Hyperth Off J Eur Soc Hyperthermic Oncol North Am Hyperth Group. 2022;39:1115–23. 10.1080/02656736.2022.2112309 36002243

[3] Couillard AB, Zlevor AM, Ziemlewicz TJ, Kisting MA, Knott E, Rossebo AE, White J, Lubner MG, Gettle LM, Hinshaw JL, Mao L. A comparison of histotripsy and percutaneous cryoablation in a chronic healthy swine kidney model. J Vasc Interv Radiol. 2023;34(11):1986–96. 37481064 10.1016/j.jvir.2023.07.014

[4] Qu S, Worlikar T, Felsted AE, Ganguly A, Beems MV, Hubbard R, et al. Non-thermal histotripsy tumor ablation promotes abscopal immune responses that enhance cancer immunotherapy. J Immunother Cancer. 2020;8:e000200. 31940590 10.1136/jitc-2019-000200 PMC7057529

[5] Worlikar T, Zhang M, Ganguly A, Hall TL, Shi J, Zhao L, et al. Impact of histotripsy on development of intrahepatic metastases in a rodent liver tumor model. Cancers. 2022;14:1612. 35406383 10.3390/cancers14071612 PMC8996987

[6] Longo KC, Zlevor AM, Laeseke PF, Swietlik JF, Knott EA, Rodgers AC, et al. Histotripsy ablations in a porcine liver model: feasibility of respiratory motion compensation by alteration of the ablation zone prescription shape. Cardiovasc Intervent Radiol. 2020;43:1695–701. 32676957 10.1007/s00270-020-02582-7 PMC8543737

[7] Smolock AR, Cristescu MM, Vlaisavljevich E, Gendron-Fitzpatrick A, Green C, Cannata J, et al. Robotically assisted sonic therapy as a noninvasive nonthermal ablation modality: proof of concept in a porcine liver model. Radiology. 2018;287:485–93. 29381870 10.1148/radiol.2018171544

[8] Smolock AR, White SB, Rilling WS, Ziemlewicz TJ, Laeseke PF, Vlaisavljevich E, et al. The development of histotripsy for the treatment of liver tumors. Adv Clin Radiol. 2022;4:137–46.

[9] Thomas GPL, Khokhlova TD, Khokhlova VA. Partial respiratory motion compensation for abdominal extracorporeal boiling histotripsy treatments with a robotic arm. IEEE Trans Ultrason Ferroelectr Freq Control. 2021;68:2861–70. 33905328 10.1109/TUFFC.2021.3075938 PMC8513721

[10] Musil P, Harsanyi S, Torok P, Paulikova M, Moens D, Kalas L, et al. Application and technical principles of catheter high-frequency jet ventilation. Adv Respir Med. 2023;91:278–87. 37489385 10.3390/arm91040022 PMC10366769

[11] Galmén K, Freedman J, Toporek G, Goździk W, Harbut P. Clinical application of high frequency jet ventilation in stereotactic liver ablations–a methodological study. F1000Research. 2018;7:773. 30271582 10.12688/f1000research.14873.1 PMC6113879

[12] Aizer A, Qiu JK, Cheng AV, Wu PB, Barbhaiya CR, Jankelson L, et al. Rapid pacing and high-frequency jet ventilation additively improve catheter stability during atrial fibrillation ablation. J Cardiovasc Electrophysiol. 2020;31:1678–86. 32314841 10.1111/jce.14507

[13] Plavka R, Dokoupilová M, Pazderová L, Kopecký P, Sebroň V, Zapadlo M, et al. High-frequency jet ventilation improves gas exchange in extremely immature infants with evolving chronic lung disease. Am J Perinatol. 2006;23:467–72. 17094040 10.1055/s-2006-954821

[14] Hautmann H, Gamarra F, Henke M, Diehm S, Huber RM. High frequency jet ventilation in interventional fiberoptic bronchoscopy. Anesth Analg. 2000;90(6):1436–40. 10825336 10.1097/00000539-200006000-00034

[15] Fritz P, Kraus H-J, Dölken W, Mühlnickel W, Müller-Nolte F, Hering W. Technical note: gold marker implants and high-frequency jet ventilation for stereotactic, single-dose irradiation of liver tumors. Technol Cancer Res Treat. 2006;5:9–14. 16417397 10.1177/153303460600500102

[16] Denys A, Lachenal Y, Duran R, Chollet-Rivier M, Bize P. Use of high-frequency jet ventilation for percutaneous tumor ablation. Cardiovasc Intervent Radiol. 2014;37:140–6. 23636246 10.1007/s00270-013-0620-4

[17] Fritz P, Kraus H-J, Mühlnickel W, Sassmann V, Hering W, Strauch K. High-frequency jet ventilation for complete target immobilization and reduction of planning target volume in stereotactic high single-dose irradiation of stage I non-small cell lung cancer and lung metastases. Int J Radiat Oncol. 2010;78:136–42. 10.1016/j.ijrobp.2009.07.1678 19910142

[18] Galmén K, Harbut P, Freedman J, Jakobsson JG. The use of high-frequency ventilation during general anaesthesia: an update. F1000Research. 2017;6:756. 28649372 10.12688/f1000research.10823.1 PMC5464224

[19] Mauch SC, Zlevor AM, Knott EA, Couillard AB, Periyasamy S, Williams EC, et al. Hepatic and renal histotripsy in an anticoagulated porcine model. J Vasc Interv Radiol. 2023;34:386-394.e2. 36503074 10.1016/j.jvir.2022.11.034 PMC11223641

[20] Cornelis FH, Durack JC, Kimm SY, Wimmer T, Coleman JA, Solomon SB, et al. A comparative study of ablation boundary sharpness after percutaneous radiofrequency, cryo-, microwave, and irreversible electroporation ablation in normal swine liver and kidneys. Cardiovasc Intervent Radiol. 2017;40:1600–8. 28516273 10.1007/s00270-017-1692-3 PMC5744668

[21] Knott EA, Longo KC, Vlaisavljevich E, Zhang X, Swietlik JF, Xu Z, et al. Transcostal histotripsy ablation in an in vivo acute hepatic porcine model. Cardiovasc Intervent Radiol. 2021;44:1643–50. 34244841 10.1007/s00270-021-02914-1

[22] Falk KL, Laeseke PF, Kisting MA, Zlevor AM, Knott EA, Smolock AR, et al. Clinical translation of abdominal histotripsy: a review of preclinical studies in large animal models. Int J Hyperthermia. 2023;40:2272065. 37875279 10.1080/02656736.2023.2272065 PMC10629829

[23] Mageras GS, Yorke E. Deep inspiration breath hold and respiratory gating strategies for reducing organ motion in radiation treatment. Semin Radiat Oncol. 2004;14:65–75. 14752734 10.1053/j.semradonc.2003.10.009

[24] Wu QJ, Thongphiew D, Wang Z, Chankong V, Yin F-F. The impact of respiratory motion and treatment technique on stereotactic body radiation therapy for liver cancer. Med Phys. 2008;35:1440–51. 18491539 10.1118/1.2839095

[25] Wagner MG, Periyasamy S, Longhurst C, McLachlan MJ, Whitehead JF, Speidel MA, et al. Real-time respiratory motion compensated roadmaps for hepatic arterial interventions. Med Phys. 2021;48:5661–73. 34431111 10.1002/mp.15187 PMC8568648

[26] Kubo HD, Hill BC. Respiration gated radiotherapy treatment: a technical study. Phys Med Biol. 1996;41:83–91. 8685260 10.1088/0031-9155/41/1/007

[27] Cavedon C. Real-time control of respiratory motion: Beyond radiation therapy. Phys Med PM Int J Devoted Appl Phys Med Biol Off J Ital Assoc Biomed Phys AIFB. 2019;66:104–12. 10.1016/j.ejmp.2019.09.241 31586767

[28] Schweikard A, Shiomi H, Adler J. Respiration tracking in radiosurgery. Med Phys. 2004;31:2738–41. 15543778 10.1118/1.1774132

[29] Pernot M, Tanter M, Fink M. 3-D real-time motion correction in high-intensity focused ultrasound therapy. Ultrasound Med Biol. 2004;30:1239–49. 15550328 10.1016/j.ultrasmedbio.2004.07.021

[30] Keall PJ, Mageras GS, Balter JM, Emery RS, Forster KM, Jiang SB, et al. The management of respiratory motion in radiation oncology report of AAPM task group 76. Med Phys. 2006;33:3874–900. 17089851 10.1118/1.2349696

[31] Warner MA, Warner ME, Buck CF, Segura JW. Clinical efficacy of high frequency jet ventilation during extracorporeal shock wave lithotripsy of renal and ureteral calculi: a comparison with conventional mechanical ventilation. J Urol. 1988;139:486–7. 3343732 10.1016/s0022-5347(17)42499-2

[32] Vlaisavljevich E, Kim Y, Owens G, Roberts W, Cain C, Xu Z. Effects of tissue mechanical properties on susceptibility to histotripsy-induced tissue damage. Phys Med Biol. 2014;59:253–70. 24351722 10.1088/0031-9155/59/2/253 PMC4888779

